# Effect of Low Temperatures upon Magnets

**Published:** 1883-08

**Authors:** 


### EFFECT OF LOW TEMPERATURES UPON MAGNETS.

A recent investigation, conducted in the physical laboratory of Harvard University, has led to the discovery of the remarkable fact that intense cold can deprive magnetized steel bars of nearly all the magnetism which may have been imparted to them. The intense cold was produced by solid carbonic acid. This fact has an important bearing upon observations of the magnetic condition of the earth taken in high latitudes; for what appear to be daily and yearly changes in the earth's magnetism may be due in large part to conditions of temperature which affect the magnets used in the observations. It also must be concluded that the molecular condition of steel is changed by great cold.

